# Updates in Pessary Care for Pelvic Organ Prolapse: A Narrative Review

**DOI:** 10.3390/jcm14082737

**Published:** 2025-04-16

**Authors:** Namrata Sethi, Ghanshyam S. Yadav

**Affiliations:** 1 Department of Obstetrics, Gynecology, and Reproductive Sciences, Yale University School of Medicine, New Haven, CT 06520, USA; namrata.sethi@yale.edu; 2 Department of Obstetrics, Gynecology and Reproductive Sciences, University of California San Diego, La Jolla, CA 92093, USA

**Keywords:** pelvic organ prolapse, pessary, pelvic floor disorders, conservative management

## Abstract

Pelvic organ prolapse (POP) affects millions of women globally, significantly impacting quality of life. Pessaries serve as a first-line, non-surgical option for symptom relief, particularly among women who wish to avoid or delay surgery. Despite widespread use, challenges persist in fitting, patient education, and long-term adherence, necessitating further advancements in design and care protocols. This narrative review was conducted to explore the role of vaginal pessaries in the management of POP, focusing on efficacy, patient adherence, complications, and emerging innovations. Pessaries demonstrate high initial success rates, but long-term adherence varies. Improper fit, discomfort, and lack of self-management contribute to discontinuation. Complications, including vaginal discharge, erosion, and bleeding, are common but generally manageable. Recent innovations, such as self-removable pessaries, 3D-printed custom designs, and hormone-releasing pessaries, show promise in improving patient experience and adherence. Studies support self-management as a cost-effective strategy that enhances patient autonomy and reduces clinic visits. Pessaries remain a valuable conservative treatment for POP, yet challenges in long-term adherence highlight the need for standardized fitting protocols, better patient education, and continued innovation in design. Future advancements should focus on patient-centered, user-friendly solutions to enhance effectiveness, comfort, and self-care, ultimately empowering women with more accessible and sustainable pelvic health options.

## 1. Introduction

Pelvic organ prolapse (POP), the protrusion of pelvic organs into the vagina due to lack of pelvic support, is a common condition. A meta-analysis of the worldwide prevalence published in 2024 found the overall pooled prevalence of POP to be 30.9%. Additionally, the prevalence of POP increases with advancing age [1]. About 50% of parous women above 50 years are affected by POP [2]. It is projected that between 4.9 and 9.2 million women in the United States will be living with POP by 2050 [3]. Risk factors include advanced age, vaginal childbirth (especially with high-birth-weight infants), obesity, chronic constipation, heavy lifting, smoking, family history of POP, and connective tissue disorders. POP affects women socially and sexually and can cause significant psychological distress. Close to 60% of women may experience a poor quality of life from prolapse [4]. Depression may affect at least 2 out of 10 women with prolapse [5].

The management of POP may be conservative or surgical, and has evolved significantly over centuries. Ancient civilizations—including the Egyptians, Chinese, and Indians—employed diverse remedies ranging from herbal concoctions to unusual substances like mold, fermented beer, and manure. The Kahun Papyrus, dating to 2000 B.C., described placing women over burning herbs to reposition prolapsed organs. Greek physicians introduced early mechanical approaches: Hippocrates recommended succussion (tipping and shaking the patient), while Polybus suggested inserting half a pomegranate into the vagina. Soranus described using linen tampons soaked in vinegar or even pieces of meat as pessaries. By the 16th century, purpose-made pessaries began to appear, with Ambrose Paré and others designing oval and globular devices tailored to the individual anatomy. Pessaries are minimally invasive silicone devices that have been the cornerstone of the non-surgical management of POP, in addition to pelvic floor muscle exercises, and have come a long way since the name was first coined from the Greek word “pessós”, meaning oval stone. Historically, oval stones have been used to prevent conception among camels by inserting them into their uteri [6].

Contraindications to pessary use include active infections such as vaginitis or pelvic inflammatory disease [7], latex allergy (relevant for latex-containing devices), non-compliance, and inability to follow up. Severe vaginal stenosis, mesh erosion, and vaginal ulceration are additional concerns [8]. Sexual activity is debated: nearly half of urogynecologists consider it a contraindication [9], particularly with space-filling pessaries like the Gellhorn [10]. Patients using a Gellhorn pessary may believe that it influences their sexual lives [11]. However, Lamers et al. [12] argue that sexual activity should not preclude pessary use, noting comparable sexual function to surgical patients.

Pessaries are commonly offered as a first-line treatment option by physicians [13]. A proportion of 98% of urogynecologists in the United States reported that they offered pessaries for prolapse, out of which 77% reported that they use it as a first-line treatment option [9]. A systematic review [14] found that providers demonstrated adequate knowledge and recommended a pessary in up to 98% cases, especially when the patient had an anterior vaginal wall defect or a contraindication to surgery. Despite the popularity of its use, the clinical consensus statement published by the American Urogynecologic Society (AUGS) and Society of Urologic Nurses and Associates (SUNA) mentioned that pessary fitting and maintenance and the management of related complications have not been standardized due to lack of evidence [15]. The statement provides standardized guidance on vaginal pessary use for POP, addressing inconsistent practices and limited high-quality evidence. It highlights safety as a central concern, focusing on regular follow-up, individualized care, and early identification of complications. It also emphasizes proper hygiene, patient counseling, and the use of vaginal estrogen where appropriate to minimize adverse outcomes.

Rantell et al. [16] identified a lack of information about the relevant and required training and education (for healthcare professionals and patients) for pessary provision, use, and management. A study performed in France [17] to investigate knowledge and current practices among healthcare practitioners (HCPs), half of whom were Ob/Gyns, stated that HCPs would welcome additional training to improve knowledge and practices. The need for training was 65% among general practitioners. Having specialist and pessary management training were found to be some factors that influenced a better attitude towards pessaries among HCPs in a systematic review [14].

We aim to perform a narrative review on pessary use, nuances in its care, surveillance, and newer advances occurring in this space.

## 2. Materials and Methods

An electronic literature search was conducted using PubMed/MEDLINE, Embase, and Cochrane Library databases for articles published between January 2000 and March 2025. Keywords and MeSH terms used included “pelvic organ prolapse”, “vaginal pessary”, “pessary fitting”, and “pessary complications”. More than a hundred articles were considered. Relevant clinical guidelines, consensus statements, and review articles were also examined to support a comprehensive narrative synthesis of current evidence and expert opinion.

## 3. Efficacy

Pessaries are known to be effective in certain groups of patients. A prospective study demonstrated a significant decrease in quantitative POP-Q measurements (Ba, Bp, and C), although it included 50 women [18]. Pessary use generally has a high satisfaction rate among patients. The satisfaction rate associated with the Gellhorn pessary or ring with a support pessary was reported as 79% in a retrospective study [19] and more than 90% in a prospective study [20]. In the latter, there was an improvement in prolapse symptoms in 90% and urinary symptoms in 58–93% of the cases. In their latest consensus statement, AUGS and SUNA recommended that all patients with symptomatic POP be offered a pessary as part of their initial treatment options [15].

A prospective study [21] found that surgery helped more in reducing the severity of prolapse symptoms than pessary use in women with Stage 2 POP or higher. Another study [22] showed no significant differences in Quality-Adjusted Life Years (QALYs) between the two treatments, and non-inferiority of pessary therapy could not be established on the Patient Global Impression of Improvement (PGI-I) scale. While pessaries were deemed a cost-effective first-line option, the study was based only on Dutch costs and reimbursement systems. In addition, a non-inferiority randomized clinical trial among 440 patients comparing pessary vs. surgery in symptomatic POP found a similar result for subjective improvement among both groups: pessary therapy as the initial strategy did not meet the criteria for non-inferiority compared with surgery for patient-reported improvement at 24 months. However, the study acknowledges the large crossover from pessary therapy to surgery as a limitation [23].

### Crossover to Surgery

Although pessaries are effective in alleviating symptoms of POP, the crossover rate to surgery is high. The continuation rate after 5 years can range from 14 to 48% [12]. A study reported the cumulative probability of continued ring pessary use as 84.1% at 1 year, progressively decreasing to 33.5% at 10 years [24]. Crossover from initial pessary to eventual surgery may occur in 12 to 69% of patients [23,25,26,27,28]. Some studies report maximum crossover within 4 months [27] of initiation of pessary use, while others have reported this to be within 2 years [25,26,29]. Pessaries may also be used among patients awaiting surgery [17,25,30] and are a handy alternative in patients who are bad surgical candidates due to comorbidities [31].

It is estimated that >200,000 POP surgeries are performed each year in the US [32]. Many women initially prefer conservative management due to concerns about complications, costs, or medical comorbidities [33]. A prospective study on 680 women reported that 63% of women with symptomatic prolapse initially opted for a pessary [34,35]. Older or menopausal women are more likely to opt for a pessary [36], whereas sexually active status [36] and younger age [25] are associated with choosing surgery.

A retrospective cohort study [27] looked at long-term adherence to pessary use among more than 700 patients. The median duration of pessary use was a little over 3 years. A total of about 17% of the patients were either tired or generally dissatisfied by pessary usage. A proportion of 28% discontinued pessary usage due to unspecified factors. This highlights that individual counseling and considering patient goals is very important when offering a pessary.

## 4. Fitting

Various pessaries are available for use, including ring, Gellhorn, shelf, cube, donut, Schaatz, Gehrung, Hodge, inflatable, and Marland pessaries, with different mechanisms of action and degrees of support. Ring pessaries are most commonly used [24,37]. According to a study on differences in position and orientation in situ between ring and Gellhorn pessaries, ring pessaries have a smaller diameter and are positioned more posteriorly vis-à-vis the inferior pubic point. This was seen on MRI using an anatomic 3D reference system [38].

Multiple aspects of optimal fit have been described, including being able to place a finger between the pessary and the vaginal wall [39], effective prolapse support, and retention during the Valsalva maneuver. The authors suggest that patient comfort ultimately guides placement and fitting. While no standardized criteria exist, clinical experience and judgment is key. Different pessary types serve specific clinical needs. Ring pessaries, with or without support, are commonly used for mild to moderate prolapse and are relatively patient-friendly. Gellhorn and cube pessaries provide stronger support for advanced prolapse but often require provider management due to difficulty with self-care. Donut pessaries suit women with wide vaginal introitus and severe prolapse. Incontinence dishes or rings with knob pessaries help manage stress urinary incontinence. Gehrung pessaries are ideal for posterior compartment defects like rectocele. Selection depends on prolapse severity, anatomy, patient comfort, ability for self-care, and the need to accommodate activities like intercourse or avoid erosion.

Making sure that the patient has the right size of the right kind of pessary to achieve the best fit is the first and foremost step in ensuring the continuation of pessary use and, hence, the success of the pessary in managing the patient’s symptoms. Improper fit may be the most common reason for discontinuation [40]. In a study conducted by Ma et al. [29], once successful pessary fitting was achieved, the continuation rate was 75% at the 5-year follow-up. In addition, being able to predict the likelihood of successful pessary use would help in clinical decision making and counseling [8].

Fitting usually has a high success rate, with 73–83% of patients with advanced or symptomatic POP having a successful pessary fitting [20,41]. Regardless, there have been numerous studies on the factors contributing to successful or unsuccessful fitting. Among the factors found to contribute to unsuccessful fitting were a higher BMI [42,43], large genital hiatus/shorter vaginal length [8,20,41,43,44], prior surgery (hysterectomy/reconstructive surgery) [8,20,42,43,44,45], de novo stress urinary incontinence (SUI) [43], sexual activity [41], advanced stage of prolapse [42,44,45], and posterior compartment prolapse [8,19,43,44].

It must be noted that these factors have not been found to contribute consistently to the success of fitting. For example, a cohort study that evaluated factors associated with pessary dislodgement in women with advanced pelvic organ prolapse stated that 16.1% of women experienced dislodgement of their ring pessaries by 6 months of use, and these women were significant for apical prolapse [46]. A meta-analysis [42] found that genital hiatus and previous hysterectomy were not associated with an unsuccessful fit. It also concluded that age was not a contributor, whereas another study [43] found it to be a contributor. Hence, fittings may vary from patient to patient even if they have a similar history or pelvic exam findings, and thus, we advise shared decision making between the patient and the provider.

Among women with advanced POP and repeated expulsion of the pessary, a perineal suturing technique has been described as a way of maintaining the pessary [47]. Three independent, loose, absorbable stitches were taken to help reduce the introitus and reinforce the perineal body. The mean age was higher in this group (75.3 vs. 68.3 years) than that in the group not undergoing this intervention. The study concluded that the standardization of perineal suturing for elderly/frail women would be helpful, as it improves their quality of life while avoiding surgery.

## 5. Complications and Discontinuation

Pessaries are, overall, safe for use. The most common adverse effects are vaginal bleeding, discharge, ulcerations, and erosions. Bleeding usually occurs in 8% to 33% of cases [40,48,49], discomfort in up to 79% [49], and erosion in about 7% to 45% [29,40,48,50]. Vaginal discharge ranges from about 15% to 25% [48,51], but may even go up to 30% [15]. It needs to be remembered that although increased vaginal discharge is common, it is often not from a vaginal infection [15].

The AUGS–SUNA consensus statement [15] recommends using the categories “erythema” (redness), “abrasion” (superficial injury, scant bleeding), “erosion/ulceration” (deeper injury, bleeding requiring cauterization) and “fistula” (vesicovaginal or rectovaginal) to classify pessary-related vaginal epithelial changes. Granulation tissue can develop from pessary use due to any of these epithelial changes. Reinsertion of the pessary is appropriate in case of erythema or abrasion; usually, no modification of care is needed. In case of erosion/ulceration, silver nitrate or Monsel’s solution may be used to achieve hemostasis. Vaginal estrogen may be tried along with continued pessary use in milder cases. Worsening erosions warrant a 4-week pessary break while using vaginal estrogen. Vaginal biopsy may be required in persistent cases. Another acceptable way of describing vaginal epithelial changes associated with pessary use is as follows [52]: no abnormalities (0), epithelial erythema (1), granulation tissue (2), epithelial break or erosion </= 1 cm (3), and epithelial break or erosion > 1 cm (4). Table 1 summarizes the four categories of vaginal epithelial changes as recommended by the consensus statement, as well as the general management for each category in practice.

A systematic review of rare complications of pessary use found that vesicovaginal fistula, rectovaginal fistula, vaginal impaction, and vaginal evisceration of small bowel through vaginal vault were the ones usually reported among rare complications [53]. According to a recent systematic review of vesicovaginal and rectovaginal fistulae associated with pessary use for POP [54], the prevalence of fistulae from pessary use is likely <1%, but the use of the Gellhorn pessary or neglect in pessary care may lead to a higher prevalence (up to 3%). Another systematic review highlighted that most complications among patients stemmed from neglect in pessary care [7]. The mean age of the patients involved was close to 79 years, which could explain neglect associated with limited mobility or cognitive decline. This reiterates that although pessaries are effective, they require careful patient selection, routine follow-up, and standardized monitoring protocols to prevent complications.

Discontinuation rates for pessary use vary widely, ranging from 8.7% to 37.6% at 1 year [11,24,29]. Difficulty with self-care, TVL < 7.5 cm, and poor urinary symptom relief at 3 months were reported as potential risk factors for discontinuation in a study [29]. The discontinuation rate went from 8.7% at 1 year to 2.7% at 5 years, suggesting that patients who are happy with the pessary continue to use it. A retrospective study [19] reported that insertion/removal difficulties and erosions were the major factors affecting dissatisfaction and discontinuation. Erosion was also one of the most common reasons for the discontinuation of pessary use (45.3%) in another retrospective study [45]. Gellhorn and donut pessaries were linked to higher erosion rates than ring pessaries, with recurrent but not single erosions contributing to discontinuation [40]. Among hundreds of women who were advised and counseled regarding self-care in a long-term study [24], the leading cause of discontinuation (21.6%) was frequent expulsion, followed by vaginal erosion, lack of prolapse improvement, and challenges in self-care.

Pain is a significant part of the experience and must be considered. In fact, among patients who decide not to use a pessary at all, the decision may be supported by the fear of pain in addition to the fear of vaginal discharge, irritation, and bleeding [14]. An evaluation of pain scores during pessary insertion and removal [55] revealed that pessary removal was more painful than insertion in 58.2% of the cases, and the difference was significant. There was also a difference between ring and other pessary types, ring pessaries being significantly less painful than shelf and Gellhorn pessaries during both removal and insertion. Increased pain, especially during removal, may be experienced in cases of smaller genital hiatus and the presence of vaginal atrophy or vulvar skin disease [51]. A clinical trial comparing lidocaine hydrochloride (HCl) 2% jelly vs. lubricating jelly for pain management during pessary removal or reinsertion during office visits reported that both groups demonstrated low pain during pessary removal and reinsertion, with no significant difference among them [56].

Some patients with pessaries may be at a higher risk of vaginal bleeding from atrophy due to age [40]. Oftentimes, a postmenopausal woman using a pessary may present with bleeding. In that case, a speculum examination is recommended to look for erosions or abrasions [57]. If erosions are present, a break in pessary use is advised, with daily estrogen application for 2 weeks. If the bleeding is consistent, another source may be considered and evaluated using a vaginal ultrasound.

## 6. Surveillance and Follow-Up

While pessaries are acceptable to patients as a management option, there seems to be no clear consensus for follow-up intervals. The optimization of follow-up times should help avoid the unnecessary use of resources while also preventing complications.

A randomized controlled trial [58] analyzed pessary complications and patient satisfaction in two different time interval groups for pessary replacement among women with POP of all stages using ring pessaries. No statistically significant difference in the complication rate was found among the 6-monthly group vs. the 3-monthly group. The patient satisfaction scores were also similar. Another randomized controlled trial [52] involving office-based pessary care and a follow-up of 48 weeks found the frequency of 24 weeks for follow-up to be non-inferior to 12 weeks; this was primarily based on the occurrence of vaginal erosions. Similarly, a cross-sectional study found no increase in complication rates when ring pessaries were cleaned and monitored every 6 months [59]. This was followed by the publication of a randomized clinical trial [60] comparing the occurrence of complications with cleaning and gynecological examination every 3 vs. 6 months of ring pessary use. The prevalence of ulcerations was similar in the two groups, but the group with cleaning every 6 months showed a higher prevalence of bacterial vaginosis.

Additionally, a retrospective study [48] was conducted to evaluate time intervals between pessary maintenance visits after the COVID-19 pandemic began, as non-emergent office visits were suspended, and whether these impacted outcomes. The median time to an in-person visit was 4.5 months. No significant association was found between the time interval between pessary visits and adverse outcomes. However, this was contradicted by another retrospective cohort study [61] that reported a significant increase in bleeding/ulceration/infection in the case of ring pessaries, with increased follow-up intervals linked to the pandemic.

Given all of the above, AUGS recommends the initial visit after pessary fitting to be scheduled within 4 weeks so that the challenges encountered by the patients may be addressed, and maintenance visits for clinic-based care to be scheduled every 1–6 months. More frequent visits would be less cost-effective, and there is a lack of evidence for an improved success rate. AUGS does, however, recommend a closer follow-up for cube pessaries in particular, due to the higher likelihood of vaginal erosions and bleeding [15]. We advise that providers tailor pessary care to each patient’s individual needs but have scheduled visits every 1–6 months.

### Self-Management

Several studies have explored the self-management of pessaries (removal and reinsertion by patients) to optimize resources and reduce complications through more frequent pessary changes. Self-management appears to support continued pessary use, with the inability to do so identified as a risk factor for discontinuation [29]. The ability to self-manage has been reported as a strong predictor of prolonged use (3–5 years) [62]. A prospective cohort study [63] found that 45% of patients who were educated about self-management could self-manage after one year, with no serious adverse events. The pain, discharge, and irritation scores remained stable at 3 and 12 months. Lower rates of vaginal bleeding have been reported in self-care patients even with less frequent follow-ups (4–6 months vs. 1–3 months in non-self-care) [64]. In this study, self-care was more common in younger, sexually active patients with less advanced POP. The “TOPSY” trial [35] suggested that women who felt confident in self-managing their pessaries not only were more likely to experience less pessary-related complications (possibly due to more frequent changing) and more likely to perceive an improvement, but they also relied less on clinic-based care, making self-management a more cost-effective option through reduced resource utilization. Older studies [65] also mention that teaching women to manage their pessaries helps reduce healthcare costs, and also takes up less clinician time.

Self-management also enhances patients’ sense of autonomy, improving their overall experience. A study on long-term outcomes [11] found self-management of the Gellhorn pessary to be safe, effective, and supportive of autonomy. Additionally, a study [66] reported that women valued self-management for fostering independence and allowing their bodies periodic rest. Daily self-replacement can reduce discharge symptoms and inflammation but does not affect the microbiota composition [67].

As per AUGS–SUNA [15], maintenance visits for women who self-manage their pessaries are recommended every 6–12 months. No timeline has been proven to be superior in this case, either. Though there are some studies that have been performed with different kinds of pessaries, the Gellhorn, shelf, and cube pessaries are designed to support the prolapse by exerting a suction effect and can be quite difficult to self-manage.

## 7. Concomitant Pessary Care

Vaginal estrogen is safe for use in pessary patients [68]. Several studies have employed the use of estrogen in various forms (cream/tablet/ring) as a way to manage complications like abrasions, erosions, or bleeding, or as part of routine pessary care [29,40]. The benefit of estrogen as a regular part of pessary use has also been evaluated in the literature. It was found [69] that the use of vaginal estrogen with pessaries for POP in postmenopausal women was associated with a lower occurrence of bacterial vaginosis (BV) and improved urinary urgency and frequency. However, estrogen use did not significantly reduce ulcerations, itching, vaginal discharge, or odor. A systematic review [70] found a significantly lower incidence of BV among postmenopausal women with POP pessaries who used estrogen vs. those who did not. Again, no effect was found on ulceration, bleeding, or discharge. A Cochrane Review [2] attempted to analyze the effects of estrogen therapy (local and systemic) in postmenopausal women with various grades of POP. They found insufficient evidence to draw any solid conclusions on the benefits or harms of estrogen therapy alone for managing POP symptoms, but they did conclude that pessaries were associated with fewer adverse vaginal events when used in conjunction with topical estrogen, and surgery was associated with lesser postoperative UTIs when topical estrogen was used concurrently.

As stated by the International Urogynecological Consultation (IUC) on POP [16], trials to support the use of vaginal estrogen for ulceration/erosions are lacking. They also recommended that limiting the use of vaginal estrogen to those who need it (such as patients with vaginal atrophy) may simplify pessary use for patients. The AUGS-SUNA consensus [15] stated that vaginal estrogen may be considered for women who “wish to optimize” their long-term pessary use. This statement was made after having analyzed several studies that did not report a significant difference in the rate of pessary-related epithelial abrasions with vaginal estrogen use, including the study by Propst et al. [52]. They did, however, find a study that reported that women who used vaginal estrogen were less likely to discontinue pessary use or develop discharge [71]. Another study also reports an association between vaginal estrogen use and longer pessary use [72].

The association between the presence of vaginal erosions and the vaginal pH has also been evaluated [73]. Pessary users with vaginal erosions were found to have a more alkaline vaginal pH and greater bacterial diversity (more Gram-negative bacteria and fewer lactobacilli) than those without erosions or non-users. No significant changes in symptoms (discharge/itching) or microbiota have been found upon investigating the impact of vaginal probiotic suppository and moisturizing vaginal gel use among postmenopausal pessary users [74]. This study was underpowered to detect a difference. Currently, there are no guidelines suggesting the routine use of probiotics in postmenopausal pessary users, as the benefits are unclear.

Hydroxyquinoline gel, a vaginal acidifier, has been studied for at least a decade, for its potential benefit in pessary users in maintaining a normal vaginal pH and reducing vaginal odor. A randomized trial [75] found no impact on BV development or symptoms within the first three months, and thus the evidence to support its use is lacking [15]. A secondary analysis of a randomized trial [76] showed increased anaerobes in women removing pessaries less than once a week, but no difference in vaginal symptoms or pessary satisfaction was noted. A cross-sectional study [77] analyzed swabs from 40 women who wore their ring pessaries continuously for at least 12 weeks, and found no association between the phenotype of the bacterial biofilm (lactobacillus dominance or deficiency) that forms on vaginal pessaries and the symptoms/signs of vaginal inflammation. A cytokine analysis in pessary users has also been performed in the literature. Significantly elevated pro-inflammatory cytokines have been found in those with vaginal epithelial abnormalities vs. those without [78]. Given that there is no consensus on how pessary use impacts the microbiome and the flora of the vagina, the role of concomitant agents to reduce the associated complications is debatable.

## 8. New Dimensions

Modern-day pessaries made of silicone have not undergone much evolution in design since they were introduced in the second half of the 20th century. They are not sufficiently patient-friendly for self-management. Innovation in this space has been lacking until recently. A recent editorial [79] reported that there has been a recent surge in innovations in pessary care, with 3D printed molds being used to create customized silicone pessaries [80], to pessaries that are designed to facilitate self-management. It was hypothesized that 3D printing using artificial intelligence and patient-specific anatomy would help because this would eliminate the trial-and-error aspect of fitting [80]. All patients reported either an improvement or no change in pessary ease of use, comfort, and the feeling of support provided by the pessary. However, this was a pilot feasibility study with a 3-week follow-up, and only eight women were included in the study.

In another study [81], an estriol-releasing pessary (with similar mechanical strength as commercial options) was developed using 3D molds to possibly allow patients to avoid estrogen creams altogether because of the need for frequent application. Different concentrations of estriol (1%, 10%, and 15%) were incorporated into the pessary for steady release and were tested. It was found, at 3 months, that these pessaries maintained their strength/were durable.

There have been some recent developments in the biomechanical space as well. Roshanfar et al. [82] proposed a new base shape design that can be adjusted as per the anatomical measurements of each patient to decrease the overall displacement of the pessary—possibly leading to more effective symptom improvement—and, thus, higher compliance in pessary use and avoidance of surgery. Another research study [83] identified hyperelastic models that could be used to understand the behavior of silicone pessaries under load and improve their biomechanical analysis. They also predict that these models could be used in the future to evaluate size/design changes in pessaries.

A few newer pessaries have also been introduced to the market in recent years. ProVate (ConTIPI Medical) [84] is a self-inserted, single-use disposable device, which was designed to perform like a ring pessary by lifting up vaginal walls. It was concluded to be more user-friendly given the use of an applicator to deploy the pessary, thus enabling self-care among women and consequently being expected to lower discontinuation rates. It was also found to be as effective as ring pessaries (the control group), with comparably low numbers of adverse events, although this study had a short follow-up period (the minimum being 33 usage days). In addition, given its disposable nature, the environmental impact of the device needs to be considered. The same authors also studied [85] whether their device clinically affects the vaginal microflora, as compared with a commercially available ring pessary. It was concluded that the device is comparable to ring pessaries in terms of the effect on the vaginal microflora.

Reia (Reia, LLC) [86] is another newer pessary available in the United States. It is collapsible and seems to be patient-friendly, with lower pain scores and an improved comfort level, thus also having a positive influence on self-care. The effectiveness and safety assessed at 3 months were comparable to those of the control pessaries (Gellhorn/ring). The pain was reported to be significantly lower during both insertion and removal.

There was a randomized trial [87] on 40 women to compare the efficacy of internal vs. external pessaries in postmenopausal women. External pessaries were meant to be worn as a brace, made up of an adjustable support, a tampon-like holder, and a silicone cushion. Internal pessaries were found to be more effective in terms of anterior and apical prolapse POP-Q scores at 3 months. However, the complication rate (bleeding, discharge, displacement, etc.) was also higher with internal pessaries. The external pessaries improved the quality of life to an equal degree as the internal pessaries.

With the growing focus on innovation in pelvic health, the objective is to develop a pessary that prioritizes patient comfort and ease of use and facilitates self-management. By enhancing accessibility and usability, this pessary shall aim to empower patients to take control of their pelvic health while maintaining effectiveness and safety while decreasing healthcare utilization. Incorporating patient feedback and ergonomic considerations, the goal is to create a solution that improves adherence, minimizes discomfort, and aligns with the evolving needs of women seeking non-invasive management options for pelvic floor disorders.

## 9. Conclusions

Although our review methodology allowed us to explore numerous aspects related to pessary selection, use, and care and highlight gaps in the existing literature, the authors acknowledge that narrative reviews tend to be prone to bias and subjectivity.

Nevertheless, pessaries remain a cornerstone in the conservative management of pelvic organ prolapse, offering an effective, non-surgical option for symptom relief. Despite their widespread use, challenges in fitting, patient education, and long-term adherence persist. Recent innovations, including self-management strategies, customizable designs, and novel materials, aim to improve user experience and compliance. Standardized training for healthcare providers and clearer surveillance guidelines are needed to optimize outcomes and minimize complications. As advancements continue, patient-centered designs that prioritize ease of use and comfort will be crucial in enhancing pessary adoption and effectiveness, ultimately empowering women with better options for managing pelvic floor disorders.

## Figures and Tables

**Table 1:** Recommendations for managing pessary-related vaginal epithelial changes. Clinicians should assess the vaginal epithelium for these changes during office visits.

Complication	Description	Suggested Management	Special Considerations
Erythema	Redness without visible epithelial breakdown	- Assess that the size and fit are correct and reinsert the pessary - Patient contacts provider if bleeding develops	
Abrasion	Superficial injury (partial thickness) with scant bleeding	Assess that the size and fit are correct and reinsert the pessary	Consider vaginal estrogen
Erosion/Ulceration	Deeper injury (full thickness) with more bleeding	- Milder cases: vaginal estrogen (determine the frequency and duration); continued pessary use - Worsening cases: pessary break; follow-up after 4–6 weeks, and re-evaluate and consider reinsertion of pessary if improved - Persistent cases: vaginal biopsy (erosion may be obscuring a precancerous/cancerous lesion)	- May switch to a smaller size or different type of pessary - May require chemical cauterization in case of bleeding - Strict close follow-up needed if pessary is reinserted after pessary break and vaginal healing
Fistula	Injury to adjacent organs: rectum or bladder	- Pessary removal and fistula management; consider recurrence risk with pessary reinsertion - Certain cases with ingrown impacted pessaries could require removal in the operating room	Pursue diagnostic testing to determine location, extent, and nature of the fistula; referral to specialist

## Data Availability

No new data were created or analyzed in this study. Data sharing is not applicable to this article.

## Abbreviations

The following abbreviations are used in this manuscript:

POP	Pelvic organ prolapse
AUGS	American Urogynecologic Society
SUNA	Society of Urologic Nurses and Associates
HCP	Healthcare practitioner
QALYs	Quality-Adjusted Life Years
PGI-I	Patient Global Impression of Improvement
IUC	International Urogynecological Consultation

## References

[1] Hadizadeh-Talasaz Z., Khadivzadeh T., Mohajeri T., Sadeghi M. (2024). Worldwide Prevalence of Pelvic Organ Prolapse: A Systematic Review and Meta-Analysis. Iran. J. Public Health.

[2] Taithongchai A., Johnson E.E., Ismail S.I., Barron-Millar E., Kernohan A., Thakar R. (2023). Oestrogen Therapy for Treating Pelvic Organ Prolapse in Postmenopausal Women. Cochrane Database Syst. Rev..

[3] Wu J.M., Hundley A.F., Fulton R.G., Myers E.R. (2009). Forecasting the Prevalence of Pelvic Floor Disorders in U.S. Women: 2010 to 2050: 2010 to 2050. Obstet. Gynecol..

[4] Tefera Z., Temesgen B., Arega M., Getaneh T., Belay A. (2023). Quality of Life and Its Associated Factors among Women Diagnosed with Pelvic Organ Prolapse in Gynecology Outpatient Department Southern Nations, Nationalities, and Peoples Region Public Referral Hospitals, Ethiopia. BMC Women’s Health.

[5] Peinado Molina R.A., Martínez Vázquez S., Martínez Galiano J.M., Rivera Izquierdo M., Khan K.S., Cano-Ibáñez N. (2024). Prevalence of Depression and Anxiety in Women with Pelvic Floor Dysfunctions: A Systematic Review and Meta-Analysis. Int. J. Gynaecol. Obstet..

[6] Oliver R., Thakar R., Sultan A.H. (2011). The History and Usage of the Vaginal Pessary: A Review. Eur. J. Obstet. Gynecol. Reprod. Biol..

[7] Calles Sastre L., Almoguera Pérez-Cejuela B., Pereira Sánchez A., Herrero Gámiz S., Magrina J.F., Ríos Vallejo M., Pérez Medina T. (2023). Complications of Pessaries Amenable to Surgical Correction: Two Case Reports and a Systematic Review of the Literature. J. Pers. Med..

[8] Turel Fatakia F., Pixton S., Caudwell Hall J., Dietz H.P. (2020). Predictors of Successful Ring Pessary Use in Women with Pelvic Organ Prolapse. Aust. N. Z. J. Obstet. Gynaecol..

[9] Cundiff G., Weidner A., Visco A., Bump R., Addison W. (2000). A Survey of Pessary Use by Members of the American Urogynecologic Society. Obstet. Gynecol..

[10] Shah S.M., Sultan A.H., Thakar R. (2006). The History and Evolution of Pessaries for Pelvic Organ Prolapse. Int. Urogynecol. J. Pelvic Floor. Dysfunct..

[11] Chien C.-W., Lo T.-S., Tseng L.-H., Lin Y.-H., Hsieh W.-C., Lee S.-J. (2020). Long-Term Outcomes of Self-Management Gellhorn Pessary for Symptomatic Pelvic Organ Prolapse. Female Pelvic Med. Reconstr. Surg..

[12] Lamers B.H.C., Broekman B.M.W., Milani A.L. (2011). Pessary Treatment for Pelvic Organ Prolapse and Health-Related Quality of Life: A Review. Int. Urogynecol. J..

[13] Bugge C., Adams E.J., Gopinath D., Stewart F., Dembinsky M., Sobiesuo P., Kearney R. (2020). Pessaries (mechanical Devices) for Managing Pelvic Organ Prolapse in Women. Cochrane Database Syst. Rev..

[14] Vasconcelos C.T.M., Silva Gomes M.L., Ribeiro G.L., Oriá M.O.B., Geoffrion R., Vasconcelos Neto J.A. (2020). Women and Healthcare Providers’ Knowledge, Attitudes and Practice Related to Pessaries for Pelvic Organ Prolapse: A Systematic Review. Eur. J. Obstet. Gynecol. Reprod. Biol..

[15] American Urogynecologic Society, Society of Urologic Nurses and Associates (2023). Vaginal Pessary Use and Management for Pelvic Organ Prolapse. Urogynecology.

[16] Rantell A., Abdool Z., Fullerton M.E., Gedefaw A., Lough K., Miotla P., Mukhtarova N., Neumann P., Spencer J., Warner K.J. (2025). International Urogynecology Consultation Chapter 3 Committee 1—Pessary Management. Int. Urogynecol. J..

[17] Pizzoferrato A.-C., Nyangoh-Timoh K., Martin-Lasnel M., Fauvet R., de Tayrac R., Villot A. (2022). Vaginal Pessary for Pelvic Organ Prolapse: A French Multidisciplinary Survey. J. Women’s Health.

[18] Mendes L.C., Bezerra L.R.P.S., Bilhar A.P.M., Neto J.A.V., Vasconcelos C.T.M., Saboia D.M., Karbage S.A.L. (2021). Symptomatic and Anatomic Improvement of Pelvic Organ Prolapse in Vaginal Pessary Users. Int. Urogynecol. J..

[19] Yang J., Han J., Zhu F., Wang Y. (2018). Ring and Gellhorn Pessaries Used in Patients with Pelvic Organ Prolapse: A Retrospective Study of 8 Years. Arch. Gynecol. Obstet..

[20] Zhou Y., Sun T., Ju A., Zhu L. (2024). Outcomes of Pessary Fitting Trials for Patients with Stage IV Pelvic Organ Prolapse: A Prospective Study. Int. Urogynecol. J..

[21] Coolen A.-L.W.M., Troost S., Mol B.W.J., Roovers J.-P.W.R., Bongers M.Y. (2018). Primary Treatment of Pelvic Organ Prolapse: Pessary Use versus Prolapse Surgery. Int. Urogynecol. J..

[22] Ben Â.J., van der Vaart L.R., Bosmans J.E., Roovers J.-P.W.R., Lagro-Janssen A.L.M., van der Vaart C.H., Vollebregt A. (2024). Cost-Effectiveness of Pessary Therapy versus Surgery for Symptomatic Pelvic Organ Prolapse: An Economic Evaluation alongside a Randomised Non-Inferiority Controlled Trial. BMJ Open.

[23] van der Vaart L.R., Vollebregt A., Milani A.L., Lagro-Janssen A.L., Duijnhoven R.G., Roovers J.-P.W.R., van der Vaart C.H. (2022). Effect of Pessary vs. Surgery on Patient-Reported Improvement in Patients with Symptomatic Pelvic Organ Prolapse: A Randomized Clinical Trial: A Randomized Clinical Trial. JAMA.

[24] Manchana T. (2024). Long-Term Continuations Rate of Ring Pessary Use for Symptomatic Pelvic Organ Prolapse. Arch. Gynecol. Obstet..

[25] Patnam R., Sripad A.A., Dengler E., Geller E.J., Wu J.M. (2020). Moving on: How Many Women Opt for Surgery after Pessary Use for Prolapse?. Female Pelvic Med. Reconstr. Surg..

[26] Dykes N., Lim Y.N., Zilberlicht A., Dwyer P.L. (2023). Are Older Patients with Prolapse Likely to Continue Pessary Use? A Retrospective Observational Study. Int. Urogynecol. J..

[27] Koch M., Carlin G., Lange S., Umek W., Krall C., Bodner-Adler B. (2023). Long-Term Adherence to Pessary Use in Women with Pelvic Organ Prolapse: A Retrospective Cohort Study. Maturitas.

[28] Ruffolo A.F., Lallemant M., Aurore D., Kerbage Y., Rubod C., Cosson M. (2024). Self-Care of Vaginal Pessary for Pelvic Organ Prolapse: A Systematic Review and Meta-Analysis. Arch. Gynecol. Obstet..

[29] Ma C., Zhou Y., Kang J., Zhang Y., Ma Y., Wang Y., Tian W., Xu T., Liang S., Fan G. (2021). Vaginal Pessary Treatment in Women with Symptomatic Pelvic Organ Prolapse: A Long-Term Prospective Study: A Long-Term Prospective Study. Menopause.

[30] Bugge C., Hagen S., Thakar R. (2013). Vaginal Pessaries for Pelvic Organ Prolapse and Urinary Incontinence: A Multiprofessional Survey of Practice. Int. Urogynecol. J..

[31] Anantawat T., Manonai J., Wattanayingcharoenchai R., Sarit-apirak S. (2016). Impact of a Vaginal Pessary on the Quality of Life in Women with Pelvic Organ Prolapse. Asian Biomed..

[32] Brown H.W., Hegde A., Huebner M., Neels H., Barnes H.C., Marquini G.V., Mukhtarova N., Mbwele B., Tailor V., Kocjancic E. (2022). International Urogynecology Consultation Chapter 1 Committee 2: Epidemiology of Pelvic Organ Prolapse: Prevalence, Incidence, Natural History, and Service Needs. Int. Urogynecol. J..

[33] Trowbridge E.R., Northington G.M. (2023). Success after Treatment of Pelvic Organ Prolapse with Surgery or Pessary Remains a Patient-Centered Choice. JAMA Surg..

[34] Kapoor D.S., Thakar R., Sultan A.H., Oliver R. (2009). Conservative versus Surgical Management of Prolapse: What Dictates Patient Choice?. Int. Urogynecol. J. Pelvic Floor. Dysfunct..

[35] Hagen S., Kearney R., Goodman K., Best C., Elders A., Melone L., Dwyer L., Dembinsky M., Graham M., Agur W. (2023). Clinical Effectiveness of Vaginal Pessary Self-Management vs. Clinic-Based Care for Pelvic Organ Prolapse (TOPSY): A Randomised Controlled Superiority Trial. EClinicalMedicine.

[36] Ko P.-C., Lo T.-S., Tseng L.-H., Lin Y.-H., Liang C.-C., Lee S.-J. (2011). Use of a Pessary in Treatment of Pelvic Organ Prolapse: Quality of Life, Compliance, and Failure at 1-Year Follow-Up. J. Minim. Invasive Gynecol..

[37] de Albuquerque Coelho S.C., de Castro E.B., Juliato C.R.T. (2016). Female Pelvic Organ Prolapse Using Pessaries: Systematic Review. Int. Urogynecol. J..

[38] Hong C.X., Meer E., Cioban M., Tischfield D.J., Hassani D.B., Harvie H.S. (2022). Position and Orientation of Vaginal Pessaries in Situ on Magnetic Resonance Imaging. Int. Urogynecol. J..

[39] Panman C.M.C.R., Wiegersma M., Kollen B.J., Burger H., Berger M.Y., Dekker J.H. (2017). Predictors of Unsuccessful Pessary Fitting in Women with Prolapse: A Cross-Sectional Study in General Practice. Int. Urogynecol. J..

[40] Kakkar A., Reuveni-Salzman A., Bentaleb J., Belzile E., Merovitz L., Larouche M. (2023). Adverse Events Associated with Pessary Use over One Year among Women Attending a Pessary Care Clinic. Int. Urogynecol. J..

[41] Vasconcelos C.T.M., Gomes M.L.S., Geoffrion R., Saboia D.M., Bezerra K.d.C., Vasconcelos Neto J.A. (2020). Pessary Evaluation for Genital Prolapse Treatment: From Acceptance to Successful Fitting. Neurourol. Urodyn..

[42] de Albuquerque Coelho S.C., Brito L.G.O., de Araujo C.C., Juliato C.R.T. (2020). Factors Associated with Unsuccessful Pessary Fitting in Women with Symptomatic Pelvic Organ Prolapse: Systematic Review and Metanalysis. Neurourol. Urodyn..

[43] Manzini C., Morsinkhof L.M., van der Vaart C.H., Withagen M.I.J., Grob A.T.M. (2022). Parameters Associated with Unsuccessful Pessary Fitting for Pelvic Organ Prolapse up to Three Months Follow-up: A Systematic Review and Meta-Analysis. Int. Urogynecol. J..

[44] Ma C., Xu T., Kang J., Zhang Y., Ma Y., Liang S., Zhu L. (2020). Factors Associated with Pessary Fitting in Women with Symptomatic Pelvic Organ Prolapse: A Large Prospective Cohort Study. Neurourol. Urodyn..

[45] Oh S., Namkung H.R., Yoon H.Y., Lee S.Y., Jeon M.J. (2022). Factors Associated with Unsuccessful Pessary Fitting and Reasons for Discontinuation in Korean Women with Pelvic Organ Prolapse. Obstet. Gynecol. Sci..

[46] Coelho S.C.A., Giraldo P.C., de Castro E.B., Brito L.G.O., Juliato C.R.T. (2021). Risk Factors for Dislodgment of Vaginal Pessaries in Women with Pelvic Organ Prolapse: A Cohort Study: A Cohort Study. Female Pelvic Med. Reconstr. Surg..

[47] Pérez-Febles M., De-Miguel-Manso S., García-García E., López-País M., Cuaresma-González M., Ibañez-Nieto M. (2023). Pessary with Perineal Suture for Treatment of Pelvic Organ Prolapse: Description and Benefit of the Technique. Arch. Gynecol. Obstet..

[48] Halani P.K., Gelman E., Duchein Y., Roselli N., Leegant A. (2022). Adapting to Challenging Circumstances: Pessary Care in a Racially Diverse Urban Population within a U.S. Epicenter of the COVID-19 Pandemic. Female Pelvic Med. Reconstr. Surg..

[49] Ramanujam S., Balakrishnan S. (2023). The Usage of Gellhorn Pessary in Pelvic Organ Prolapse and in Regards to Success, Continuity of Use and Effect on Symptoms: A Retrospective Study of 2 Years. Med. J. Malays..

[50] Li B., Chen Q., Zhang J., Yu C., Zhang L., Chen L. (2020). A Prospective Study of Pessary Use for Severe Pelvic Organ Prolapse: 3-Year Follow-up Outcomes. Arch. Gynecol. Obstet..

[51] Kruyt L.M., van der Ploeg J.M., Lammers K., van Etten-Debruijn B.A., Niemeijer A.S., Hakvoort R.A. (2024). Pessaries for Pelvic Organ Prolapse: Evaluation of Vaginal Discharge and Pain during Pessary Cleaning in an Outpatient Setting. Int. Urogynecol. J..

[52] Propst K., Mellen C., O’Sullivan D.M., Tulikangas P.K. (2020). Timing of Office-Based Pessary Care: A Randomized Controlled Trial. Obstet. Gynecol..

[53] Dabic S., Sze C., Sansone S., Chughtai B. (2022). Rare Complications of Pessary Use: A Systematic Review of Case Reports. BJUI Compass.

[54] Curtis T.J., Chant C., Quek S., Giarenis I., Gray T.G. (2025). Fistulae Secondary to Vaginal Pessary Use for Pelvic Organ Prolapse: A Systematic Review. Int. Urogynecol. J..

[55] Renouf C., Ballard P., Khunda A., Kershaw V., Shawer S., Rees J. (2024). Patient Experience of Pain during Vaginal Pessary Removal and Insertion: A Service Evaluation Study. Int. Urogynecol. J..

[56] Jackson A.A., Frisco S.C., Lynch C.M., Tanner J.P., Propst K. (2024). Topical Lidocaine for Pessary Removal and Reinsertion Pain Reduction: A Randomized Clinical Trial. Am. J. Obstet. Gynecol..

[57] Dueñas J.L., Miceli A. (2018). Effectiveness of a Continuous-Use Ring-Shaped Vaginal Pessary without Support for Advanced Pelvic Organ Prolapse in Postmenopausal Women. Int. Urogynecol. J..

[58] Tam M.-S., Lee V.Y.T., Yu E.L.M., Wan R.S.F., Tang J.S.M., He J.M.Y., Lui L.K.Y., Chiu K.-P., Cheung R.Y.K., Lee K.-W. (2019). The Effect of Time Interval of Vaginal Ring Pessary Replacement for Pelvic Organ Prolapse on Complications and Patient Satisfaction: A Randomised Controlled Trial. Maturitas.

[59] de Albuquerque Coelho S.C., Pereira G.M.V., Brito L.G.O., Juliato C.R.T. (2022). Cross Sectional Study on Assessment of Ring Pessary Cleaning and Removal Every Six Months: Adverse Events and Complications. Int. Urogynecol. J..

[60] Giorgenon G.V., Galhardo L.M., de Araujo C.C., de Castro E.B., Brito L.G.O., Juliato C.R.T. (2024). Complications in Pelvic Organ Prolapse with 3-Month versus 6-Month Pessary Care: Pilot Study. Urogynecology.

[61] McNeill E.R., Lucocq J., Brown K., Kay V. (2023). The Impact on Complication Rates of Delayed Routine Pessary Reviews during the COVID-19 Pandemic. Int. Urogynecol. J..

[62] Manonai J., Sarit-Apirak S., Udomsubpayakul U. (2018). Vaginal Ring Pessary Use for Pelvic Organ Prolapse: Continuation Rates and Predictors of Continued Use. Menopause.

[63] Thys S.D., Hakvoort R.A., Asseler J., Milani A.L., Vollebregt A., Roovers J.P. (2020). Effect of Pessary Cleaning and Optimal Time Interval for Follow-up: A Prospective Cohort Study. Int. Urogynecol. J..

[64] Holubyeva A., Rimpel K., Blakey-Cheung S., Finamore P.S., O’Shaughnessy D.L. (2021). Rates of Pessary Self-Care and the Characteristics of Patients Who Perform It. Female Pelvic Med. Reconstr. Surg..

[65] Kearney R., Brown C. (2014). Self-Management of Vaginal Pessaries for Pelvic Organ Prolapse. BMJ Qual. Improv. Rep..

[66] Dwyer L., Rajai A., Dowding D., Kearney R. (2024). Understanding Factors That Affect Willingness to Self-Manage a Pessary for Pelvic Organ Prolapse: A Questionnaire-Based Cross-Sectional Study of Pessary-Using Women in the UK. Int. Urogynecol. J..

[67] Yoshimura K., Morotomi N., Fukuda K., Kubo T., Taniguchi H. (2020). Changes of Intravaginal Microbiota and Inflammation after Self-Replacement Ring Pessary Therapy Compared to Continuous Ring Pessary Usage for Pelvic Organ Prolapse: Ring Pessary and Intravaginal Microbiome. J. Obstet. Gynaecol. Res..

[68] Biehl C., Plotsker O., Mirkin S. (2019). A Systematic Review of the Efficacy and Safety of Vaginal Estrogen Products for the Treatment of Genitourinary Syndrome of Menopause. Menopause.

[69] de Albuquerque Coelho S.C., Giraldo P.C., Brito L.G.O., Juliato C.R.T. (2021). ESTROgen Use for Complications in Women Treating Pelvic Organ Prolapse with Vaginal PESSaries (ESTRO-PESS)—A Randomized Clinical Trial. Int. Urogynecol. J..

[70] Ai F., Wang Y., Wang J., Zhou L., Wang S. (2022). Effect of Estrogen on Vaginal Complications of Pessary Use: A Systematic Review and Meta-Analysis. Climacteric.

[71] Dessie S.G., Armstrong K., Modest A.M., Hacker M.R., Hota L.S. (2016). Effect of Vaginal Estrogen on Pessary Use. Int. Urogynecol. J..

[72] Wolff B., Williams K., Winkler A., Lind L., Shalom D. (2017). Pessary Types and Discontinuation Rates in Patients with Advanced Pelvic Organ Prolapse. Int. Urogynecol. J..

[73] Bouchard M.-E., Rousseau E., Fortier L.-C., Girard I. (2021). Pathophysiology of Vaginal Erosions in Women Using Pessary: A Pilot Study Examining Vaginal Microbiota. J. Obstet. Gynaecol. Can..

[74] Sappenfield E.C., Mellen C., Wilcox J., O’Hanlon D.E., O’Sullivan D.M., Tunitsky-Bitton E. (2024). The Impact of Vaginal Probiotics on Pessary Use: A Randomized Controlled Trial. Urogynecology.

[75] Meriwether K.V., Rogers R.G., Craig E., Peterson S.D., Gutman R.E., Iglesia C.B. (2015). The Effect of Hydroxyquinoline-Based Gel on Pessary-Associated Bacterial Vaginosis: A Multicenter Randomized Controlled Trial. Am. J. Obstet. Gynecol..

[76] Fregosi N.J., Hobson D.T.G., Kinman C.L., Gaskins J.T., Stewart J.R., Meriwether K.V. (2018). Changes in the Vaginal Microenvironment as Related to Frequency of Pessary Removal. Female Pelvic Med. Reconstr. Surg..

[77] Gould F.G., Carey M.P., Plummer E.L., Murray G.L., Danielewski J.A., Tabrizi S.N., Garland S.M. (2022). Bacterial Biofilm Formation on Vaginal Ring Pessaries Used for Pelvic Organ Prolapse. Int. Urogynecol. J..

[78] Ramaseshan A.S., Mellen C., O’Sullivan D.M., Nold C., Tulikangas P.K. (2022). Host Inflammatory Response in Women with Vaginal Epithelial Abnormalities after Pessary Use. Int. Urogynecol. J..

[79] Kisby C. (2023). A New Dimension in Pessary Care. Urogynecology.

[80] Hong C.X., Zhang S., Eltahawi A., Borazjani A., Kalami H., San A.N., Sham D., Ameri G., McDermott C.D. (2023). Patient-Specific Pessaries for Pelvic Organ Prolapse Using Three-Dimensional Printing: A Pilot Study. Urogynecology.

[81] Long J., Zidan G., Seyfoddin A., Tong S., Brownfoot F.C., Chowdary P. (2022). An Estriol-Eluting Pessary to Treat Pelvic Organ Prolapse. Sci. Rep..

[82] Roshanfar M., Fatehi E., Torkaman T., Ashouri N., Lalani I., Khademi S., Aghili M., Saboukhi A., Gangal M. Toward Patient-Specific Pessary to Manage Pelvic Organ Prolapse: Design and Simulation. Proceedings of the 2023 45th Annual International Conference of the IEEE Engineering in Medicine & Biology Society (EMBC).

[83] Wanuch K.M., Blokker A., Kalami H., Hong C.X., McLachlin S.D. (2024). Hyperelastic Material Models for Simulating Deformation of Silicone Ring Pessaries. J. Mech. Behav. Biomed. Mater..

[84] Ziv E., Erlich T. (2023). A Randomized Controlled Study Comparing the Objective Efficacy and Safety of a Novel Self-Inserted Disposable Vaginal Prolapse Device and Existing Ring Pessaries. Front. Med..

[85] Ziv E., Keller N., Erlich T. (2024). Vaginal Microflora Following the Use of a Disposable Home-Use Vaginal Device and a Commercially Available Ring Pessary for Pelvic Organ Prolapse Management: A Randomized Controlled Trial. Arch. Gynecol. Obstet..

[86] Strohbehn K., Wadensweiler P.M., Richter H.E., Grimes C.L., Rardin C.R., Rosenblatt P.L., Toglia M.R., Siddiqui G., Hanissian P. (2024). Effectiveness and Safety of a Novel, Collapsible Pessary for Management of Pelvic Organ Prolapse. Am. J. Obstet. Gynecol..

[87] Hosoume R.S., Peterson T.V., Soares Júnior J.M., Baracat E.C., Haddad J.M. (2024). A Randomized Clinical Trial Comparing Internal and External Pessaries in the Treatment of Pelvic Organ Prolapse in Postmenopausal Women: A Pilot Study. Clinics.

